# Notes and News

**Published:** 1903-05-23

**Authors:** 


### NOTES AND NEWS.

HOSPITAL MEETINGS AND FESTIVALS.

Hospital for Sick Children, Great Ormond Street.—Annual Meeting, Monday, May 25, at 5 p.m.

North-Eastern Hospital for Children.—Annual Meeting, Thursday, May 28, at 4.30 p.m.

Hospital for Consumption, Brompton.—Annual Meeting, Thursday, May 28, at 4 p.m.

Should it be wet on the 22nd inst. the reception at Marlborough House by their Royal Highnesses the Prince and Princess of Wales of the ladies and gentlemen connected with the League of Mercy will not take place.

A statistical report has recently been issued by a special committee of the Glasgow Lunacy District Board on the question of alcoholic insanity. The committee has had under its consideration the patients admitted during the year ended May 15th, 1902, to the Glasgow District Asylums at Woodilee and Gartloch, and to the observation wards of Barnhill Poorhouse. Particulars of each case were obtained and an analysis compiled therefrom. From the table thus drawn up it appears that of 565 admissions to the two asylums as insane, and 213 to the observation wards of the poorhouse as cases of incipient insanity during the 12 months, no fewer than 259, or 33 per cent., have become chargeable to the parish through alcoholic indulgence. In the large majority of these cases the patients' surroundings were good, and they had been in receipt of good wages. These results bear out what is a most noticeable feature of the annual reports of asylums not only in the Glasgow district but throughout the United Kingdom, namely, the large percentage of lunatics whose insanity is due to alcoholic excess. Though it is probable that the percentage is higher north of the Tweed, yet it is considerable in all parts of the United Kingdom. The committee is of opinion that the question calls for the earnest consideration of the Government and the public at large, though it refrains from suggesting a remedy.

Lord Iveagh has offered a donation amounting to nearly £40,000 to Trinity College, Dublin, in connection with the building and equipment of scientific laboratories.

We are informed that an important meeting will be held during the first week in June at the Vichy State Springs, Paris, to inaugurate the opening of a new wing to the establishment, at which ceremony the English Ambassador will be present.

Several cases of small-pox have occurred in the Harrow district. The patients have been removed to the Small-pox Hospital, South Mimms, High Barnet, and the strictest precautions have been taken to prevent the spread of the disease in the Harrow neighbourhood.

The Medical, Surgical, and Hygienic Exhibitors Association, Limited, will hold their seventh annual exposition of professional exhibits at the Queen's Hall, Langham Place, London, W., on June 2nd, 3rd, 4th, and 5th, from 2 p.m. to 10 p.m. each day. There will be music by a select orchestra every afternoon and evening. Nurses in uniform will be admitted.

There seem to be great differences of opinion as to the value of the alien immigrant among those who have had ample opportunities of observing his habits in the various parts of the United Kingdom. So far as the inquiry into the question has gone at present it would seem that as he arrives in this country the average alien is not a person to be admired, but from some of the evidence given before the Commission one is led to hope that under favourable conditions he may be transformed into a valuable and law-abiding citizen.

Sir Robert Anderson presided at the ninety-sixth annual meeting of the Aged Pilgrims' Friend Society, held in the Mansion House on May 6th. There was a large attendance of supporters and friends. Mr. J. E. Hazelton (secretary) gave an abstract report of the Society's work, and stated that the work of the Society is divided into two branches; a providing of life pensions of five, seven, and ten guineas per annum, and of four Homes in which 178 of the recipients have peaceful and comfortable dwellings. No fewer than 1,616 pensioners, living in all parts of the kingdom, from the Shetland Isles to Cornwall, and from South-West Ireland to East Anglia, are now upon the books, and the total annual sum expended in pensions is £12,152. These figures are the highest yet reported, and show an increase over last year of 37 pensioners and £364 in pensions. The pensioners are graded as follows:—217 are on the ten guinea pension, 1,256 on the seven guinea, and 143 on the five guinea. The ages of the recipients range from 60 to 95, 1,180 being over 70 years of age, and of this number, 426 are over 80, and 30 more than 90. Of the pensioners 717 live in London, and 901 in the provinces; 13 per cent. of them are men. During the 96 years of the Society's existence, more than 7,480 aged pilgrims have received life pensions, amounting in the aggregate to upwards of £330,500. During the past year the subscriptions amounted to £4,850. More annual subscribers of 7s., 10s., and 14s., are especially desired.

